# Current Progress in Breast Implant-Associated Anaplastic Large Cell Lymphoma

**DOI:** 10.3389/fonc.2021.785887

**Published:** 2022-01-06

**Authors:** Yichen Wang, Qi Zhang, Yufang Tan, Wenchang Lv, Chongru Zhao, Mingchen Xiong, Kai Hou, Min Wu, Yuping Ren, Ning Zeng, Yiping Wu

**Affiliations:** Department of Plastic and Cosmetic Surgery, Tongji Hospital, Tongji Medical College, Huazhong University of Science and Technology, Wuhan, China

**Keywords:** BIA-ALCL, epidemiology, genetic predisposition, bacterial contamination, implant modification

## Abstract

Breast implant-associated anaplastic large-cell lymphoma (BIA-ALCL) is an uncommon type of T-cell lymphoma. Although with a low incidence, the epidemiological data raised the biosafety and health concerns of breast reconstruction and breast augmentation for BIA-ALCL. Emerging evidence confirms that genetic features, bacterial contamination, chronic inflammation, and textured breast implant are the relevant factors leading to the development of BIA-ALCL. Almost all reported cases with a medical history involve breast implants with a textured surface, which reflects the role of implant surface characteristics in BIA-ALCL. With this review, we expect to highlight the most significant features on etiology, pathogenesis, diagnosis, and therapy of BIA-ALCL, as well as we review the physical characteristics of breast implants and their potential pathogenic effect and hopefully provide a foundation for optimal choice of type of implant with minimal morbidity.

## 1 Introduction

Over the past few decades, breast implants have been widely used worldwide for breast augmentation and breast reconstruction. Breast implant-associated anaplastic large cell lymphoma (BIA-ALCL) is a variant of anaplastic large cell lymphoma (ALCL) that presents with seroma effusion associated with breast implants, particularly those with a textured outer shell. In 1997, Keech and Creech reported the first case of BIA-ALCL with silicone breast implants (1). Subsequently, emerging studies have reported more than 800 cases of this uncommon lymphoma disease in women with breast implants (2, 3). In 2008, a case-control study in the Netherlands initially found the association between breast implants and ALCL (4). The World Health Organization (WHO) included BIA-ALCL as a clinicopathologic entity for systemic/nodular and lymph node lymphoma into the fourth edition of the Lymphoma Classification of WHO in 2017 (5).

Nowadays, BIA-ALCL is attracting more attention with the increasing number of cases. However, the exact pathogenesis of BIA-ALCL remains relatively poorly understood. Considering that most cases of BIA-ALCL are diagnosed in patients with textured implants, it is implied that the texture or surface roughness of the implant is related to the pathogenesis of this uncommon disease. In this review, we expect to highlight the most significant features on etiology, pathogenesis, diagnosis, and therapy of BIA-ALCL as well as we review the physical characteristics of breast implants and their potential pathogenic effect. The comprehensive understanding of BIA-ALCL is critical for early recognition and timely surgical resection.

## 2 Clinical Features

BIA-ALCL is an uncommon lymphoproliferative disease. Although BIA-ALCL shares some similar morphological and immunophenotypic characteristics with other anaplastic lymphoma kinase-negative ALCL, its manifestations and clinical process are closely related to the implantation of breast implants. To date, all reported cases with a detailed implant history are involved in textured surface breast implants (6). The time interval from implantation to the diagnosis of BIA-ALCL varies ranging from 2 years to 32 years at the latest, with a median interval of 8-10 years. The most common clinic pathological characteristic of BIA-ALCL is the effusion or persistent seroma around the implants. Some BIA-ALCL patients present with a tumor mass with or without effusion (7). Some patients have related regional lymph node involvement, usually axillary lymph node swelling, and the 5-year overall survival of patients with lymph node involvement is significantly worse (8). Alcalá et al. reported that a 56-year-old woman with BIA-ALCL accompanied by several papules on her right breast skin (9). Histologic examination of the skin nodules showed proliferation of lymphocytes with irregular shapes and polymorphic nuclei, indicating skin involvement as the first manifestation of BIA-ALCL. Notably, Bautista-Quach et al. reported the first case of bilateral BIA-ALCL after bilateral breast implantation in 2013 (10). Pathological examination showed that ALCL involved both breast implant capsules with subclinical symptoms that appeared on unilateral breast.

Besides, Laurent et al. investigated that BIA-ALCL was a unique clinical entity consisting of two histological subtypes depended on clinical characteristics: *in situ* BIA-ALCL, the effusion around the implant, anaplastic cell proliferation confined to the fibrous capsule; infiltrative BIA-ALCL, the palpable mass penetrating adjacent tissue and sometimes resembling Hodgkin lymphoma (7). It was presumed that *in situ* BIA-ALCL possessed a more moderate clinical course and generally could be relieved after implant removal, but infiltrative BIA-ALCL could have a more malignant clinical course and might require additional therapy with implant removal. Other investigators consider the variable clinical and pathologic as part of the spectrum of the disease and its progression over time (2).

## 3 Epidemiology

In the past 20 years, many studies support that BIA-ALCL is a unique lymphoid malignant tumor and its incidence rates vary greatly across the world. Most of the existing reported BIA-ALCL occurred in Europe and America, including the United Kingdom, the United States, Italy, Netherlands, and Australia, while BIA-ALCL is extremely rare in the population of Asian, African, and Native American descent (2). Four consecutive BIA-ALCL cases in Asia have been reported in 2020 and 2021, suggesting that the true incidence of BIA-ALCL in Asia may be underestimated. Furthermore, it is interesting that Srinivasa et al. performed a database query and evaluations in 40 countries/regions, in which 363 cases of unique ALCL associated with breast augmentation were reported (11). All implant manufacturers have ALCL cases related to implants. Thus, there is an evidential link between textured breast implants and BIA-ALCL. It is needed to emphasize the awareness and vigilance of the public, medical professionals, and regulatory agencies on BIA-ALCL.

In a prospective study, 4 out of 17,656 patients who received Natrelle 410 (Allergan) breast implants in the USA eventually developed BIA-ALCL, indicating the BIA-ALCL incidence actually might be close to 1 per 4000 cases (12). Doren et al. conducted a retrospective study in the USA and estimated an incidence rate was 2.03 per 1 million person-years in textured BIA-ALCL (13). Cordeiro et al. conducted a prospective cohort study in a total of 3546 patients who underwent breast reconstruction at a large cancer center in the USA (14). About 10 women developed BIA-ALCL after a median exposure of 11.5 years, and the overall risk of BIA-ALCL was 1 in 355 women or 0.311 cases per 1000 person-years. The same group also reported that the total incidence of BIA-ALCL over 26 years was 1.79 per 1000 patients and 1.15 per 1000 implants in the USA (15). It also demonstrated that BIA-ALCL incidence might be higher than previously estimated, especially in patients with textured implants for more than 10 years. In an Italian study, Campanale et al. performed a retrospective study on BIA-ALCL cases collected in the Dispovigilance database, showing that there were 22 BIA-ALCL cases and the incidence of related Italian BIA-ALCL cases in 2015 was 2.8 per 100,000 patients (16). In the Netherlands, De Boer et al. identified 32 cases of BIA-ALCL with breast implants (17). Among women under the age of 75, one breast ALCL may occur in every 6920 women with implants.

There are only four cases reported in Asia. Ohishi et al. described the first case of BIA-ALCL discovered in Japan (18). The patient was a 67-year-old Japanese woman with breast cancer (BC) who underwent mastectomy and reconstruction with a textured silicone breast implant in 2002 and unfortunately developed ALCL in 2018. The first known case of BIA-ALCL in Thailand was a 32-year-old woman who developed BIA-ALCL after using a textured implant for breast augmentation for 3 years (19). Kim et al. reported the first case of BIA-ALCL in South Korea which showed a typically delayed seroma around the implant 7 years after the implantation of a textured implant (20). The latest report was from Taiwan, where massive periprosthetic fluid accumulation was detected in the left breast, and histological examination revealed pleomorphic neoplastic lymphoid cells (21). The detailed information of BIA-ALCL that occurred in Asia is listed in **Table 1**. These reported cases suggest that plastic surgeons in Asia need to raise awareness of delayed seroma formation and BIA-ALCL.

**Table 1:** The detailed information of BIA-ALCL occurred in Asia.

Country/region (Ref)	Implant type	Time of onset	Manifestation	Treatment
Japan (18)	Textured surface breast implant (McGhan Limited/ 410LM 220g/REF 27-LM115-220/LOT 161276	17 years after implantation	Induration and redness presented in the left breast, fluid collection around the breast implant, contralateral axillary lymphadenopathy, CD30 (+) and ALK (-) cells	Breast implant was removed along with as much surrounding capsule as possible, excisional biopsy of contralateral axillary lymph node, adjuvant CHOP chemotherapy
Thailand (19)	Anatomical textured silicone implant (Silimed, Rio de Janeiro, Brazil)	3 years after implantation	Swelling in the left breast for 2 weeks, periprosthetic fluid, CD30 (+) and ALK (-) cells	Bilateral removal of the implant, ipsilateral total capsulectomy, and removal of yellowish fibrinous material around the implant
South Korea (20)	Biocell silicone-filled textured breast implant (Allergan Inc., Irvine, CA).	7 years after implantation	Fluid collection surrounding the right breast implant, multiple hard, immobile masses of various sizes below the inner surface of the right breast capsule CD30 (+) and ALK (-) cells	Breast implant removal and capsule biopsy, chemotherapy and radiation therapy
Taiwan (21)	Biocell textured surface anatomical shape silicone implants (350g/LOT 2885279)	3 years after implantation	Progressive swelling of the left breast, massive periprosthetic fluid accumulation in the left breast, CD30 (+) and ALK (-) cells	Bilateral complete capsulectomy with implant

ALK, anaplastic lymphoma kinase; CHOP, cyclophosphamide doxorubicin vincristine prednisolone.

However, some studies believed that there was no clear evidence between breast implants and ALCL. In a breast implant clinical study sponsored by Allergan, Largent Joan et al. reported 3 cases of ALCL in women with breast implants and a history of BC with an overall incidence rate 1.46/100,000 person-years (22). MajaØlholm and Navin both declared that no cases of ALCL were found in their studies on women undergoing breast implant surgery (23, 24). These studies were industry-sponsored and had severe flaws in the monitoring of patients as well as in the limited time of observation. Wang et al. supported a positive correlation between breast implants and ALCL risk, but the incidence of ALCL in women with breast implants was still very low (25). Due to the rarity of BIA-ALCL, it might be necessary to include a larger sample size and longer follow-up time to explore the risk factors of ALCL.

The currently published studies on the risk of BIA-ALCL use different research methods and come from different countries and regions. Although the report shows that the absolute risk of developing BIA-ALCL is small, these figures may lack accuracy. The lack of prevalence among women with different types of implants, the lack of detailed reports of adverse events related to breast implants, poor attitudes towards the diagnosis and management of this disease, and the increasing phenomenon of beauty tourism, may lead to some missed new cases, thus underestimating the actual incidence and risk of BIA-ALCL. In recent years, the rapid increase in the risk and incidence of BIA-ALCL over time may be the result of increasing awareness of the diagnosis of this new type of pathological entity. Totally, the number of affected patients with BIA-ALCL has increased in recent years, leaving tomorrow for discussion on the effect of breast implants and their association with BIA-ALCL.

## 4 Etiology

**Figure 1:**
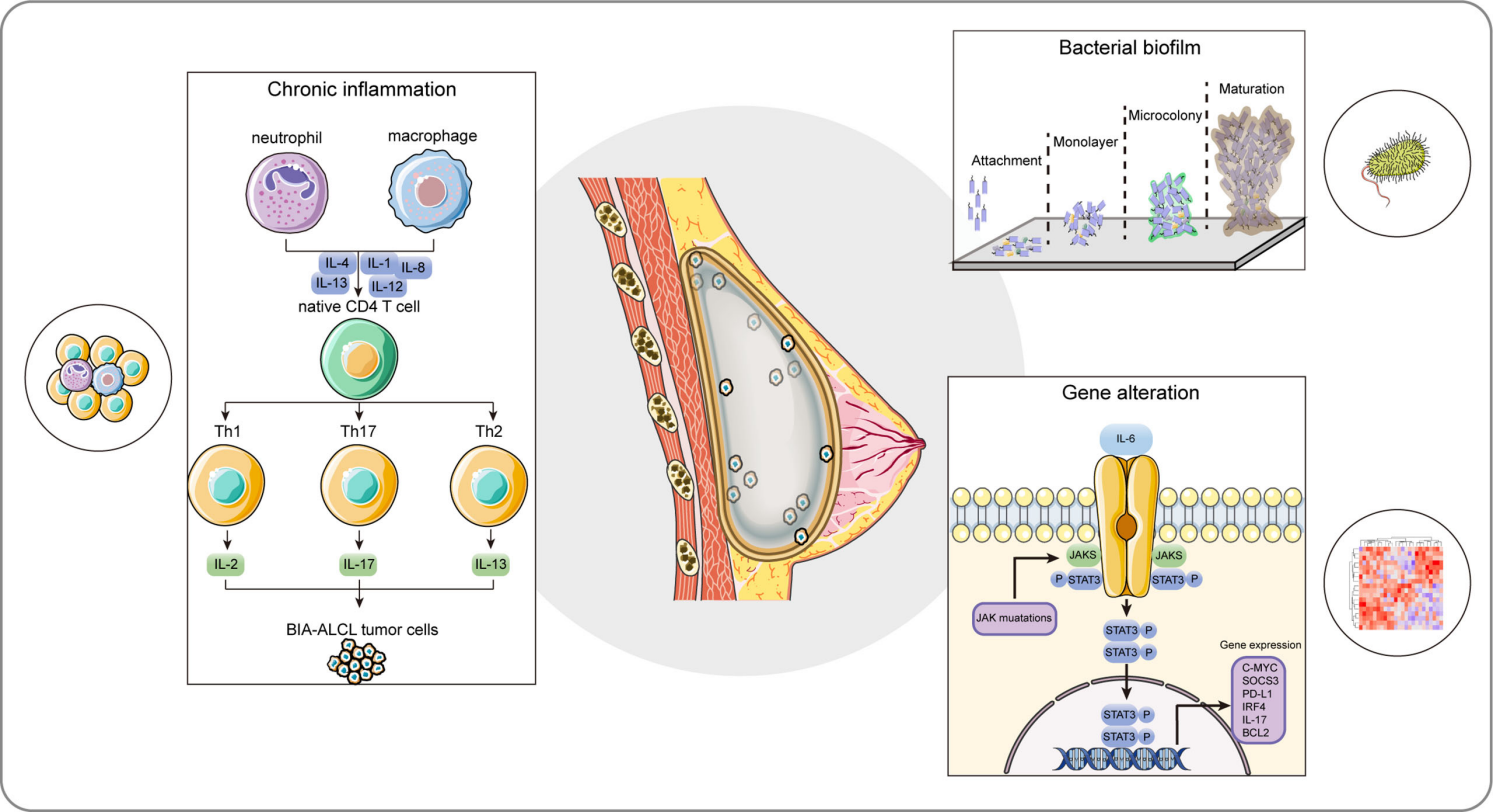
The proposed hypothesis of the cellular and molecular etiological factors for BIA-ALCL. BIA-ALCL formation involves multiple interplays of internal and external factors collectively, including genetic predisposition, bacterial biofilm, chronic inflammation, and textured breast implant. The composition of the textured implant could be identified as a foreign antigen, and the textured surface of the implant provides a proper environment for bacterial biofilm colonization, formation, and development. Cytokines produced by bacterial infection and autoimmune activate CD4+ T cells, thus leading to a persistent chronic inflammatory state and resulting in the clone reproduction of activated CD4+ T cells. The aberrantly oncogenic JAK/STAT3 pathway mutations and IL-6-induced overexpressed STAT3 signal pathway promote phenotypic differentiation of Th1/Th17 and Th2 lymphocytes. These factors together ultimately result in the uncontrolled T cell clone expansion and the formation of BIA-ALCL. BIA-ALCL, breast implant-associated anaplastic large cell lymphoma; IL-1, interleukin-1; IL-2, interleukin-2; IL-4, interleukin-4; IL-8, interleukin-8; IL-12, interleukin-12; IL-13, interleukin-13; IL-17, interleukin-17; JAK, Janus kinase; STAT3, transcription factor 3.

BIA-ALCL is a multifactorial disease with complex processes resulting from various contributing factors synergistically. The pathogenesis of BIA-ALCL is not well defined. However, several plausible mechanisms have been proposed. The pathogenesis of BIA-ALCL is still incompletely understood, but several possible mechanisms have been proposed. The prevailing hypothesis for BIA-ALCL formation, includes genetic predisposition, bacterial biofilm (BF), chronic inflammation, and textured breast implant (**Figure 1**). Comprehensive oncogenic studies in a large cohort are still needed to illustrate the pathogenesis of BIA-ALCL.

### 4.1 Genetic Features and Predisposition

BIA-ALCL possesses a specific pattern of genetic alterations. The BIA-ALCL pathogenesis involved in genetic predisposition mainly includes JAK-STAT, DNA methyltransferase 3 alpha (DNMT3A) mutation, Tumor protein p53 (TP53) mutation, programmed cell death 1 ligand 1 (PD-L1) chromosomal copy number aberrations (CNAs), chromosome 20q loss, and carbonic anhydrase 9 (CA9) overexpression. Among these biological mechanisms, the constitutive activation of JAK-STAT3 has recently been frequently explored and identified as a potential key mediator in BIA-ALCL. Abnormal JAK-STAT3 signaling pathway is essential for BIA-ALCL tumorigenesis and development, and may provide a new therapeutic target for some patients. However, to date, according to the conclusion, oncogenic JAK-STAT3 pathway mutations have been described in 43.8% of successfully tested cases (26). It indicated that JAK-STAT3 is not that specific in BIA-ALCL and other mechanisms are also very important. The complexity of pathogenesis, individual differences, sample size, and research methods result in the complexity of BIA-ALCL mechanism. Further studies in a larger cohort are needed to determine the effect of predisposing genetic factors in BIA-ALCL.

Genetic susceptibility is an important factor in the occurrence and development of BIA-ALCL. At first, Blombery et al. identified activating mutations in JAK1 and STAT3 in extracted blood and effusion of two BIA-ALCL cases (27). In their next study, they observed JAK/STAT activation in 10 patients, and identified TP53 mutations, repeated copy number loss of ribosomal protein L5 (RPL5), and high-level amplification of TNF receptor superfamily member 11a (TNFRSF11A) and platelet-derived growth factor receptor alpha (PDGFRA) (28). Similarly, Di Napoli et al. found dysregulated activation of the JAK/STAT pathway including STAT3 and suppressor of cytokine signaling 1 (SOCS1) mutations in one BIA-AlCL case, and nonsense mutations in DNMT3A in another BIA-AlCL case (29). Oishi et al. observed that in all cases, high expression of pSTAT3 was associated with JAK1 or STAT3 mutations, implying oncogenic JAK-STAT3 activation in BIA-ALCL (30). Laurent et al. provided a comprehensive genomic landscape of BIA-ALCL including JAK/STAT activating mutations and inactivation of epigenetic modifiers, involving lysine methyltransferase 2C (KMT2C), lysine methyltransferase 2D (KMT2D), chromodomain helicase DNA binding protein 2 (CHD2), and CREB binding protein (CREBBP) (31). These results emphasized the significance of the BIA-ALCL genomic landscape characterized by both JAK/STAT activating mutations and epigenetic alterations.

Li Fraumeni syndrome (LFS) is a rare cancer predisposition syndrome caused primarily germline pathogenic mutations in the TP53 gene. LFS/germline TP53 mutation might be an additional risk factor for BIA-ALCL development. In 2015, Lee et al. reported the first rare case of BIA-ALCL and LFS in a patient with germline mutation of TP53 and 13q14.3 deficiency (32). In the second report of BIA-ALCL in the setting of LFS, the patient with LFS undergoing breast implant reconstruction after BC surgery developed BIA-ALCL (33). Adlard et al. also reported a case of BIA-ALCL in a 53-year-old woman who had been diagnosed with LFS in 2019 (34).

Programmed cell death protein-1 (PD-1) has a key function to induce T cell exhaustion and tumor evasion, and the clinical values of PD-1/PD-L1 profile vary between subtypes of lymphoma. Bianchi et al. firstly provided that there was a strong expression level of PD-L1 in almost all tumor cells in the excised BIA-ALCL capsular tissue (35). The sALCL, primary cutaneous ALCL, and BIA-ALCL had similar characteristics, involving constitutive activation of the STAT3 pathway, PD-L1/PD-1 immune-checkpoint expression, PD-L1 gene amplification, and TP53 deficiency (36). Tabanelli et al. suggested that the 9p24.1 alterations represented a common mechanism of PD-L1 overexpression in the BIA-ALCL, possibly acting synergistically with constitutive pSTAT3 signaling, while PD-L1 expression might be induced by JAK/STAT signaling alone and/or other alternative pathways in PD-L1-positive cases without chromosomal aberration (37). In summary, some studies have identified frequent PD-L1 expression and recurrent PD-L1 CNAs in BIA-ALCL, suggesting that targeting the PD-1/PD-L1 axis seems to be promising in treating BIA-ALCL.

Human leukocyte antigen (HLA) polymorphisms might also increase the risk of developing BIA-ALCL. Tevis et al. discovered a difference in HLA A*26 allele frequencies in patients between BIA-ALCL patients and the normal population, which indicated a genetic predisposition factor for HLA germline genetic variation in BIA-ALCL patients (38). Identification of HLA allele differences in BIA-ALCL patients may help identify patients with textural implants at higher or lower risk of lifelong BIA-ALCL development. By using next-generation sequencing, it was found that BIA-ALCL was characterized by loss of chromosome 20q and was present in a high percentage of patients, thus distinguishing this disease from other types of ALCL (39). In addition, genome-wide CNAs analysis could be used to distinguish BIA-ALCL-induced seroma from other seroma accumulation, like infection or trauma. Oishi et al. found a remarkable up-regulation of hypoxia signal genes represented by CA9 in BIA-ALCL compared with non-BIA-ALCL (40). They also indicated that CA9 might be a potent biomarker for early diagnosis and/or long-term follow-up of BIA-ALCL. Mukhtar et al. reported a 59-year-old woman with a history of prosthetic implants suffered concurrent BIA-ALCL and invasive ductal carcinoma of the left breast (41).

### 4.2 Bacterial Contamination

BF is a mode of microorganism consortia sticked in a thick extracellular matrix (ECM), which confer protection against antimicrobials and transfer of nutrients. The formation of biofilm and its related infection are the key factors affecting the success of inserting medical devices.

Several *in vitro* studies have reported that rough-textured breast implants promoted the increase of propensity for biofilm growth compared with smooth surface implants (42). This is due to their larger surface area and increased bacterial adhesion attaching to rough surfaces. It was confirmed by James et al. that rougher breast implants with more surface (Siltex and Biocell) harbored more BF than those smoother implants (Silk and Velvet) (43). Jones et al. evaluated 11 available implants and classified them into 4 reclassified by surface area/roughness, further analysis showed a prominent positive correlation between implant surface area and bacterial attachment/growth (44). Adams et al. jointly aggregated data and evaluated the utilization of macrotextured breast implants (Biocell and polyurethane) and confirmed cases of BIA-ALCL (45). They concluded that minimizing bacterial attachment during surgery, especially for higher-risk macrotextured implants, could decrease the development of capsular contracture and BIA-ALCL.

BF is thought to be involved in the association between bacterial contamination and BIA-ALCL tumorigenesis. Hu et al. found that there was an increasing T-cell response to chronic biofilm infection around breast implants, as well as a linear relationship between the bacteria number and the proliferation of lymphocytes, which is crucial in the case of BIA-ALCL (46). In the subsequent study, they further revealed the high bacterial abundance in both BIA-ALCL and nontumor capsule patients accompanied by a different microbiome formation. Especially, the proportion of *Ralstonia* spp. present in ALCL samples was significantly higher than that in non-tumor capsule specimens (47).

Despite the associations, some scholars put forward some different views on previous research. Brody et al. was against the view that biofilms were the primary initiator of BIA-ALCL (48). He doubted that if the pathogen was *Ralstonia* spp. which was ubiquitous in many water supplies, more cases of BIA-ALCL should have been identified. Walker et al. identified the most common *Staphylococcus* spp. and *Propionibacterium* spp. were in both the BIA-ALCL and side-controlled breast (49). These results showed that there was no significant difference in bacterial microbiota diversity between BIA-ALCL and control while there was a relatively low abundance of *Ralstonia* spp., which was the opposite of the previous work (47).

### 4.3 Chronic Inflammation

BIA-ALCL represents a unique type of peripheral T-cell lymphoma. Chronic inflammation is well recognized as an underlying cause of lymphocyte transformation, lymphomagenesis, and even BIA-ALCL. Interestingly, there is evidence that chronic inflammation from BF, implant debris, and leachables might be important initiating and triggering factors in the development of BIA-ALCL. Transcriptional analysis showed that compared to normal T-cells, C-C motif chemokine ligand 18 (CCL18), C-X-C motif chemokine ligand 14 (CXCL14), and C-C motif chemokine receptor 6 (CCR6) were upregulated genes involved in leukocyte subsets migration and differentiation in BIA-ALCL (50). In particular, CCR6 was preferentially expressed by immature dendritic cells (DCs), T helper cell 17 (Th17), and regulatory T cells and it has a critical role in cellular migration to inflammatory sites. Cytokine expression profile of the BIA-ALCL cell line revealed the strong production of T cell-related cytokines including interleukin-6 (IL-6), interleukin-10 (IL-10), interferon-gamma (IFN-γ), and interleukin-2 (IL-2) (51).

BIA-ALCL occurs in an inflammatory microenvironment with significant lymphocyte and plasma cell infiltration and a prominent T helper cell 1 (Th1)/Th17 phenotype in advanced disease (52). Kadin et al. showed that BIA-ALCL possessed expression of transcription factors suppressor of cytokine signaling 3 (SOCS3), JunB proto-oncogene (JunB), special AT-rich sequence binding protein 1 (SATB1), and a Th1 phenotype-like cytokine profile (52). They proposed that the cytokine and transcription factor profiles of BIA-ALCL were in line with that BIA-ALCL was caused by the combined factors of chronic bacterial antigen stimulation, continuous T cell proliferation, and genetic predisposition (52). They also demonstrated that the amounts of interleukin-3 (IL-3) and its main transcription factor GATA binding protein 3 (GATA3) were increased in both anaplastic tumor cells and BIA-ALCL specimens (53). Besides, Interleukin-13 (IL-13)-stimulated-IgE encapsulated large numbers of eosinophils and mast cells in the affected tissues, verifying the importance of enhanced immune response characteristics of chronic allergy in the pathogenesis of BIA-ALCL.

These results support the hypothesis that the chronic inflammatory microenvironment in BIA-ALCL stimulates the immune response, induces T cell plasticity, releases inflammatory cytokines and chemotaxis, and leads to polyclonal expansion of Th17/Th1 cell subsets, ultimately leading to malignant transformation.

### 4.4 Textured Breast Implant

The implant texturing could increase implant stability on the chest wall and reduce the risk of capsular contracture after augmentation clinically. However, emerging evidence has confirmed that the majority of BIA-ALCL cases were reported in those patients implanted with textured implants, indicating that implant texturization might be a risk factor for BIA-ALCL occurrence.

Jong et al. firstly investigated that the odds ratio of BIA-ALCL in association with breast implants was 18.2 in the Netherlands (4). They continued to identify that the BIA-ALCL cases in the Netherlands were more often macroscopic textured implant (82%) based on known data (17). Tevis et al. identified 52 confirmed BIA-ALCL patients at a single academic institution in the USA, 41 of whom were exposed to implants with textured surfaces (54). In a retrospective study related to the epidemiology of BIA-ALCL, the risk of BIA-ALCL for high-texture and high-surface-area implants (grade 4 surface) was as high as 1/2,832 (3). Leberfinger et al. evaluated 304 related articles documenting BIA-ALCL and found that almost all recorded BIA-ALCL cases were associated with textured surfaces (55). These preliminary findings indicated that there was an association between textured silicone implant and ALCL. Brody et al. investigated a total of 173 cases worldwide and suggested that chronic inflammation in specific areas caused by the surface texture of silicone breast implants seemed to be the cause, and rare genetic susceptibility and biofilm organisms might be participators (11).

Some studies have verified a potential association between textured implants with different brands/models and the development of BIA-ALCL. Loch-Wilkinson et al. found that polyurethane (Silimed) and Biocell textures had a higher surface area than Siltex textures (56). Compared with Siltex textured implants, Biocell textured implants had a 14.11 times higher risk of BIA-ALCL, while Polyurethane (Silimed) textured implants had a 10.84 times higher risk of BIA-ALCL in Australia and New Zealand. Next, they further compared clinical implant exposure data and company sales for 4 distinct prostheses to calculate the implant-particular risk, and found implants with higher surface area/texture appeared to be more associated with BIA-ALCL in Australia (57). Magnusson et al. reported that the confirmed BIA-ALCL cases continued to increase and implant-specific risk changed in Australia and New Zealand (58). The BIA-ALCL risk was up-regulated for Silimed polyurethane (23.4 times higher) compared to Biocell and had increased (16.5 times higher) compared to Siltex implants, which indicated a strong relationship between implant surface area/roughness and the BIA-ALCL.

## 5 Diagnosis

### 5.1 Imaging

Current imaging methods for BIA-ALCL detection mainly include breast ultrasound (US), computed tomography (CT), magnetic resonance imaging (MRI), and positron emission tomography/computed tomography (PET/CT). Adrada et al. reported a retrospective, single-institution imaging study of BIA-ALCL, indicating that US, MRI, CT, and PET/CT possessed 84%, 82%, 55%, and 38% sensitivity for effusion detection and 46%, 50%, 50%, and 64% for mass detection, respectively. Additionally, mammography had a sensitivity of 73% and a specificity of 50% in detecting abnormalities without distinguishing effusions or masses (59). Due to the relatively insufficient understanding of unique biology and frequently non-specific appearance of ALCL, the relevant imaging test results may not be particularly ideal, suggesting that a better understanding of the imaging performance spectrum related to BIA-ALCL is needed.

The US is the first choice to assess for swelling or mass associated with breast implants (60, 61). BIA-ALCL usually exhibits a uniform effusion around the implant and inflammatory alterations in the breast tissue around the implant (59, 61, 62). In some cases, the swelling manifested as an irregular sac-like under ultrasound. Besides, among those implant-related masses, BIA-ALCL mass usually appears as an oval, hypoechoic, and well-defined solid mass. US detection can also be used to assess local area axillary or supraclavicular lymph node enlargement and guided fine needle aspiration (FNA) for cytological analysis. Breast MRI is the second imaging method to diagnose BIA-ALCL after US, especially when US produces uncertain results (63, 64). Breast MRI can accurately investigate breast implant capsule integrity/contracture, implant rupture, tissue edema, presence of fluid, and mass damage. Mammography is recommended to be routinely used for detecting high-risk and malignant disease patients. In the case of BIA-ALCL, non-specific envelope thickening, membrane contour destruction, asymmetric circumferences around the implant, or irregular mass changes (no calcification) might be observed.

CT can identify unilateral peri-implant effusion type BIA-ALCL, mass-forming BIA-ALCL, and rare bilateral BIA-ALCL, but CT has low sensitivity in detecting effusion. By CT imaging, the effusion usually manifests as non-enhanced exudation, with or without irregular implant contours or folds, which is indistinguishable from the rupture of the implant (63, 65, 66). The subtype of the mass is a diffuse lesion that partially surrounds the implant (62). The most important value of CT lies in the detection of locally advanced mass-forming BIA-ALCL (chest wall infiltration), local regional staging, and long-term staging of nodules and extranodal sites (67). Although most cases are in the early stages, PET/CT is usually recommended as the first choice to fully stage the disease (67). The National Comprehensive Cancer Network (NCCN) guideline showed that fludeoxyglucose (FDG) PET/CT could be performed for detecting associated capsular masses and chest wall involvement during the preoperative staging of local and distant diseases (60).

### 5.2 Pathological Diagnosis

BIA-ALCL can manifest as fluid accumulation around the implant and less frequently tumor mass, which are the primary objects to be examined. If delayed seroma associated with BIA-ALCL is suspected, at least 50 mL serum should be aspirated with fine needles for cytology (60). The first requirement for the diagnosis of BIA-ALCL is the specific morphological abnormality observed on standard cytology (68, 69). Cytological analysis revealed polymorphic large cells with an irregular nucleus (70). It may also display scattered or vesicular chromatin, prominent nucleoli, moderately abundant cytoplasm, and small vacuoles. The subpopulations of cells with horseshoe-shaped or kidney-shaped nuclei are called “marker cells”.

If BIA-ALCL is suspected initially in morphology, the cell block could be prepared and further analyzed by immunohistochemistry (IHC) (71). BIA-ALCL is negative for ALK like other ALCLs, and a strong and consistent positive for CD30 can provide a direction for diagnosis. Subsequently, other markers (such as T cell and B cell markers) are needed for further characterization. In contrast, many BIA-ALCLs show the expression of CD15, CD13, and CD33 bone marrow markers, which may be related to the up-regulation of genes involved in myeloid cell differentiation recently reported in BIA-ALCL. Additional biomarkers, including CD2, CD3, CD4, CD5, CD7, CD8, CD45, and ALK are also needed to be considered to establish the diagnosis and exclude other malignancies (60).

Flow cytometry (FCM) technology is the second-line assessment in the BIA-ALCL diagnostic evaluation, through the lymphocyte surface marker combination panel and forward/side scatter (FSC/SSC) to ascertain large cells (72). FCM can discriminate different types of lymphoma, carcinomas, or their coexistence, thus helping to the qualitative and quantitative assays of BIA-ALCL associated cells. Barr et al. pointed that the malignant anaplastic cells in ALCL showed strong CD30 expression, high FSC, and variable SSC, with CD4 expression and reduced/negative expression of other T-cell antigens (73). Despite the presence of reactive CD30 cells, FCM is more sensitive and specific than cytology to distinguish between BIA-ALCL and negative cases (74). Therefore, some studies also proposed that the FCM method should also be regarded as the first-line diagnostic tool.

Polymerase chain reaction (PCR) technology is mainly used to evaluate the molecular genetic mechanism in BIA-ALCL (7) (72). For those patients with breast masses or enlarged lymph nodes, histology and IHC are preferred to establish the diagnosis while PCR technology is also applicable. PCR detection of T cell receptor (TCR) gene rearrangement is the main assessment of the possible molecular genetic mechanism of BIA-ALCL. Clonal TCR gene rearrangements appear in almost all reported BIA-ALCL. However, the PCR results of TCR need to be combined with other clinical and pathological characteristics for comprehensive analysis. In addition, many recurrent mutations have been detected in BIA-ALCL, which can be used as a standard for supplementary evaluation. Among them, the most involved are mutations in JAK-STAT pathway genes, such as JAK1 and STAT3 mutations and epigenetic modifiers.

**Figure 2:**
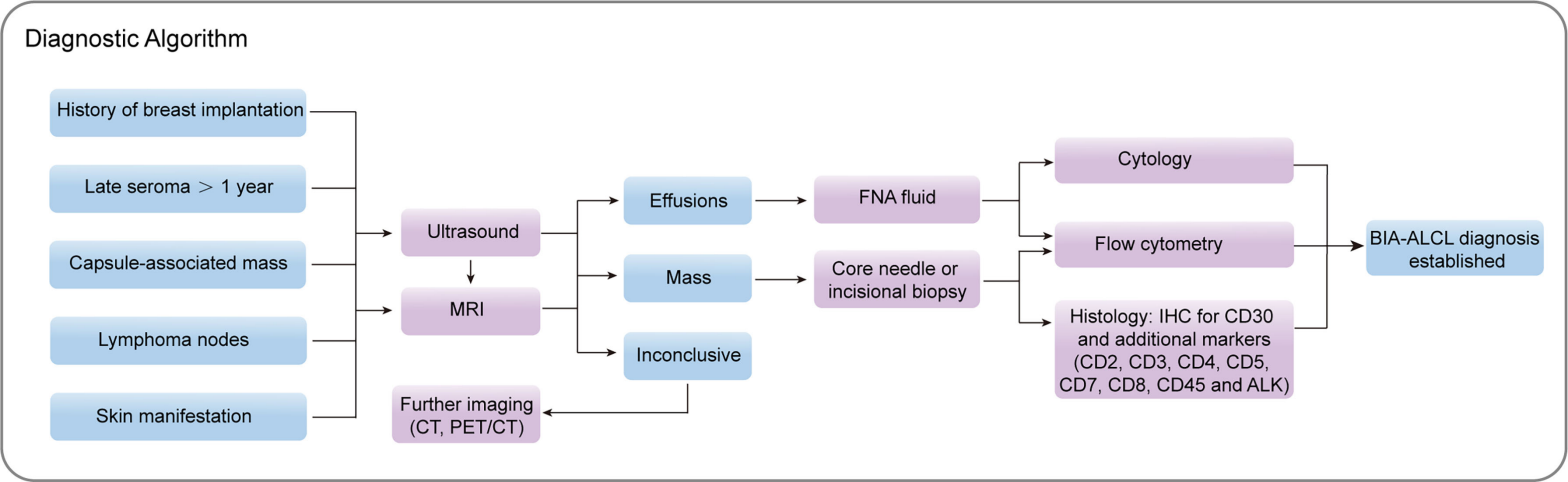
Proposed diagnostic algorithm for BIA-ALCL. MRI, magnetic resonance imaging; CT, computed tomography; PET/CT, positron emission tomography/computed tomography; FNA, fine needle aspiration; IHC, immunohistochemistry; BIA-ALCL, breast implant-associated anaplastic large cell lymphoma.

Barbé et al. shared the experience and the strategy used to diagnose BIA-ALCL: CD30 positive provided the first direction for the diagnosis of BIA-ALCL (75). If CD30-positive atypical large cells are observed, other markers such as T cell and B cell markers are needed for further identification. Histological biopsy of solid samples was the preferred diagnostic method. With the increase of serum samples, FCM technology to select large cells will be the future diagnostic trend. Jaffe et al. recommended that for effusion samples, Wright-Giemsa or other Romanowsky stains could be used to perform a cytological assessment of the seroma around the affected implant to obtain an accurate diagnosis; for cell block samples, H&E staining and IHC analysis of tissue sections were ideal, while PCR-based T cell receptor gene re-arrangement studies to detect clonality were also applicable (72). Recently, Lyapichev et al. proposed a standard pathologic procedure of every suspicious BIA-ALCL that includes pre-operative and post-operative evaluation of the case, previous fixation, pinning flat of the capsule, mapping the specimen with iconic positions, and orientation of the specimen for thorough sampling (76). Any mass or thickening on the capsule should be sampled generously, if no obvious lesions were identified, two representative slices of each of the six landmark aspects of the capsule should be taken. This approach had achieved ideal results in the detection of BIA-ALCL was used to assess the degree of disease to a certain extent. More comprehensive studies should provide sensitivity and specificity data to determine the potential value of multiple assays in combination to evaluate BIA-ALCL in clinical practice. We summarized the diagnostic algorithm of BIA-ALCL in **Figure 2**.

## 6 Therapy

### 6.1 Standard Therapy

Timely diagnosis and complete removal of the implants and surrounding fibrous capsules are the optimal treatment for most patients with BIA-ALCL (77). The purpose of surgery is to remove breast implants and any associated mass, as well as to perform an excisional biopsy of suspicious lymph nodes. Compared with other therapeutic interventions, complete surgical resection can prolong overall survival and event-free survival. Most patients with BIA-ALCL, where most effusions are confined to the fibrous capsule, could achieve complete remission through capsulectomy and implant removal (78). A small part of patients with tumor mass was more likely to present clinical aggression, and might require systemic treatment in addition to implant removal. Clemens et al. evaluate the event rate of each treatment intervention of 87 patients with pathologically diagnosed BIA-ALCL (77). Compared with patients who underwent partial capsulotomy, systemic chemotherapy, or radiotherapy, patients who underwent complete surgical resection including total capsulotomy with breast implant removal had better overall survival and event-free survival.

**Figure 3:**
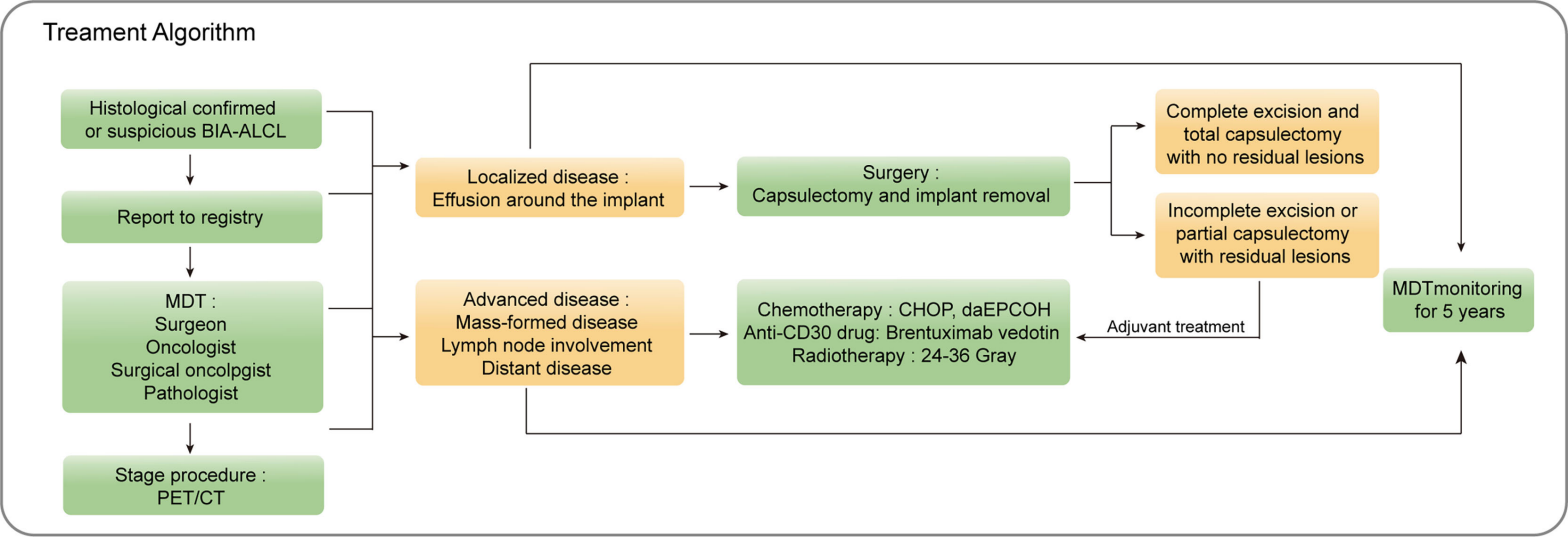
Proposed treatment algorithm for BIA-ALCL. BIA-ALCL, breast implant-associated anaplastic large cell lymphoma; MDT; multidisciplinary team; CHOP, cyclophosphamide doxorubicin vincristine prednisolone; daE, dose adjusted etoposide.

Systemic therapy is recommended for mass-formed disease, lymph node involvement, or distant disease (60, 78). For more advanced cases, individualized systemic treatment is suggested. The recommended chemotherapy option is inferred from studies about ALK-negative sALCL (6, 55, 60). The first-line treatment of sALCL is an anthracycline-based regimen (cyclophosphamide, vincristine, doxorubicin, and prednisone) with or without radiotherapy, followed by autologous stem cell rescue. A large phase III double-blind randomized trial showed that the anti-CD30 antibody drug conjugated with brentuximab vedotin could improve median progression-free survival (PFS) (79, 80). Case reports showed that the chest wall infiltration of BIA-ALCL patients was relieved when treated with brentuximab vedotin (81, 82). Anthracycline-based chemotherapy regimens (cyclophosphamide, vincristine, doxorubicin, and prednisone) or the addition of brentuximab vedotin are considered as the first-line treatment for sALCL (6). For T1 and T2 tumors with complete tissue resection, it is not recommended to routinely perform adjuvant chest wall radiotherapy after total capsulotomy (71). If complete resection is not possible, chest wall radiotherapy should be considered, or even if a complete capsulotomy is performed, the surgical margin is still positive or chest wall infiltration is present. The NCCN guidelines recommend the use of 24-36 Gray (Gy) for local or affected area radiotherapy (60). We summarized the treatment algorithm of BIA-ALCL in **Figure 3**.

### 6.2 Implant Modification

Nowadays, silicone implants are widely used in the medical field of plastic or reconstructive applications to achieve the ideal aesthetic outcome. Silicones, also known as polydimethylsiloxanes (PDMS), are commonly used implant materials with the capability of non-toxicity, softness, biological stability, chemical inertness, cell and tissue compatibility, and low cost. However, despite major advances in the design and manufacture of breast implants, it still sometimes brings some unexpected complications, such as capsular contracture, infections, and BIA-ALCL, due to roughness, inherent hydrophobicity, tissue interaction of the material. As bacterial infections and inflammation might be important factors for BIA-ALCL occurrence, the antibacterial and anti-inflammation modifications of textured implants are possible for the prevention of biofilms formation and inflammation. Here we listed some implant surface modification strategies in **Table 2**.

**Table 2:** Various strategies of silicone surface modification.

Surface modification	Molecules	Mechanism	Ref
Drug	doxycycline	inhibit *MRSA* and *Pseudomonas aeruginosa* biofilm formation and bacterial adhesion	(83)
Metal and metal oxides NPs	Ag	Inhibit the biofilm formation for *E. coli, S. aureus, Enterococcus, coagulase-negative staphylococc*i and *P. aeruginosa*	(84)
		antifungal activity of *C. albican* and biocompatible with human dermal fibroblasts	(85)
	Zn	rapid bactericidal function on *E. coli* and satisfactory biosecurity	(86)
	Cu	rapid bactericidal function on *E. coli* and satisfactory biosecurity	(86)
	ZnO	bactericidal properties both on gram-negative and gram-positive bacteria	(76, 87)
	TiO_2_	antibacterial activity against *E. coli*, photoreactivity and cell adhesiveness	(88)
	CuO	biocompatibility, antibacterial on *E. coli, S. aureus* and anticorrosive properties	(89)
GO	GO	stronger antibacterial activity against *E. coli* with thin film attributed by oxidative stress mechanism	(90, 91)
Glyco-combined nanoparticles	CMC, CMD, AA	long-lasting stability, hydrophilicity of PDMS, reduced the adsorption of negatively charged BSA and egg albumin, increased positively charged bacteriolysis	(92)
	CMC, CHI	prevent bacteria from adhering and loading and releasing antibacterial agents and anti-inflammatory agents	(93)
	HA-MKM	bacterial growth inhibition, excellent antifouling and antibacterial properties	(94)
	PLL, HA	Reduce inflammation and capsule formation	(95)
Others	PMPC	prevent non-specific protein adsorption and fibroblast adhesion to silicone surfaces	(96)
	ADM	alleviate the acute in vitro foreign body response of breast fibroblasts	(97)
	ECM	reduce the inflammation of the implant-driven foreign body response	(98)

MRSA, Methicillin-resistant Staphylococcus aureus; GO, graphene oxide; CMC, carboxymethyl cellulose; CMD, carboxymethyl β-1,3-dextran; AA, alginic acid; BSA, bovine albumin; CHI, chitosan; HA, hyaluronic acid; MKM, N^ϵ^-myristoyl-lysine methyl ester; PLL, poly-l-lysine; PMPC, 2-methacryloxyethyl phosphorylcholine; ADM, acellular dermal matrix; ECM, extracellular matrix.

#### 6.2.1 Antibiotic Treatment

The risk of BIA-ALCL could be minimized by strict operating procedures with antibacterial irrigations to reduce the chance of implant-related infections. Therefore, some researchers have proposed the use of antiseptics as a means of preventing BIA–ALCL. Barnea et al. treated the fragments on the shell of textured silicone breast implants by plasma activation combined with antibacterial irrigation with 10% povidone-iodine, cefazolin or gentamicin, which showed that surface hydrophilicity enhanced the adsorption capacity of the antibacterial irrigation (99). Culbertson et al. tested the bactericidal activity of various antibacterial breast irrigations and recommended that betadine-containing irrigation which consisted of Betadine, cefazolin, and gentamicin for breast pocket irrigation could minimize the risk of BIA-ALCL presumably by inhibiting the formation of BF (100). Besides, the specific antibiotic coating is also able to reduce the relapse and progress of capsular contracture and BIA-ALCL. The silicone breast implants coated with doxycycline on the surface inhibited *Methicillin-resistant Staphylococcus aureus (MRSA)* and *Pseudomonas aeruginosa* biofilm formation and bacterial adhesion (83). Nablo et al. established a sol-gel derived (xerogel) film capable of storing Nitric oxide (NO) and coated it on a medical-grade silicone elastomer to resist the aggressive *Staphylococcus aureus* infection in a rat model. These results indicated that NO-releasing coatings significantly reduced the incidence of biomaterial-related infections. However, the effect of antibiotic irrigation in breast augmentation has not been corroborated by other researchers. Drinane et al. conducted a cohort study and found that triple antibiotic breast irrigation did not reduce the incidence or severity of capsular contracture compared with sterile saline when using high-quality surgical technique (101). Pfeiffer et al. proposed that there was no significant difference in development of capsular contraction between patients using topical antibiotics and patients not treated with topical antibiotics (102).

#### 6.2.2 Surface Modification

Metal nanoparticles (NPs) (Ag), metal oxides NPs (ZnO, TiO2, CuO), and polymer NPs, have been widely studied for their excellent broad spectrum of antimicrobial properties to prevent biofilm formation on the implant surface. The surface modification of implants in conjunction with the above materials has been introduced to a wide range of medical coatings. Regardless of capsular contractures, nanoparticles could be ideal materials against bacterial or protein adhesions. Roe et al. developed a method of covering plastic catheters with bioactive silver nanoparticles as a coating layer (84). These catheters were biocompatible and had antibacterial properties expecting to reduce the risk of infection complications in patients with indwelling catheters. Ag NP-coated silicone elastomer had ant activity of *Candida albicans* and was biocompatible with human dermal fibroblasts *in vitro* (85). Okada et al. invented a composite material composed of titanium dioxide (TiO_2_) particles modified with amino groups on the surface and an organic silicon substrate formed by covalent bonding at the interface, and showed effective antibacterial activity against *Escherichia coli*, photoreactivity, and cell adhesiveness (88).

Noimark et al. incorporated crystal violet and bis(octyl)-phosphinic-acid-capped zinc oxide (ZnO) nanoparticles with photodynamic therapy (PDT) capability into medical-grade silicones (76). This nanoparticle endowed strong bactericidal properties both on gram-negative and gram-positive bacteria. Ozkan et al. incorporated a mixture of ZnO nanoparticles and crystal violet into PDMS through a simple two-step method (87). This modified polymer exhibits excellent hydrophobicity and significant antibacterial activity against *Escherichia coli* and *Staphylococcus aureus.* Tavakoli et al. developed a PDMS-SiO_2_-CuO hydrophobic nanocomposite coating coated on the surface of 316L stainless steel (89). This nanocomposite was promising for biomedical implants, as it exhibited favorable biocompatibility, antibacterial and anticorrosive properties. Jäger et al. synthesized Zn-doped and Cu-doped SiOx films by atmospheric pressure plasma chemical vapor deposition with rapid bactericidal function and satisfactory biosecurity (86).

Easy-to-recycle and non-inhalation hazard coatings provide an alternative to the application of antimicrobial medical devices. Graphene oxide (GO) coating has advantages in reducing possible inhalation risks compared with free-standing nanosheets. Liu et al. prepared a GO coating on a polymer substrate (90). The coatings showed stronger antibacterial activity against *Escherichia coli* with the thin film attributed by the oxidative stress mechanism. Furthermore, Gomes et al. also deposited a dispersion containing silicone rubber (SR) and Graphene nanoplatelets or its oxidized form coating on the surface of silicone by dip and spray and inhibited antimicrobial activity (91). The strong antibacterial consequence was linked with a stable and cell-compatible coating that will not delaminate from the SR surface.

Multifunctional nano-coating armed with anti-adhesion and drug delivery functions are attractive for implant modification. Yang et al. reported a method for microchannel modification of PDMS with carboxymethyl cellulose (CMC), carboxymethyl β-1,3-dextran (CMD), and alginic acid (AA) (92). The modified surface had long-lasting stability, the hydrophilicity of PDMS, reduced the adsorption of negatively charged bovine albumin (BSA) and egg albumin, and increased positively charged bacteriolysis. Park et al. reported a surface coating assembled by CMC and chitosan (CHI) through layer-by-layer assembly (LbL), which could prevent bacteria from adhering, loading and releasing antibacterial agents and anti-inflammatory agents (93). Bračič et al. formed novel nanometric layers consisting of an anionic glycosaminoglycan (hyaluronic acid (HA)) and a lysine-derived biocompatible cationic surfactant coated on PDMS (94). PDMS coated with three layers of HA- Nϵ-myristoyl-lysine methyl ester (MKM) resulted in bacterial growth inhibition, while HA enhanced the effectiveness of the incorporated surfactants, thus possessing excellent antifouling and antibacterial properties. For relieving the capsular contracture, Yoo et al. generated microtextured PDMS surfaces modified by LbL deposition of poly-l-lysine (PLL) and HA to produce a new physicochemical surface on PDMS-based silicone implants (95).

Preventing infection is an effective strategy to reduce lymphocyte activation and the risk of capsular contracture and the ultimately possible occurrence of ALCL. Park et al. used a biofilm-like polymer poly (2-methacryloxyethyl phosphorylcholine) (PMPC) to prevent non-specific protein adsorption and fibroblast adhesion to silicone surfaces (96). Compared with the uncoated implant, the capsule of the PMPC-coated silicone implant was significantly thinner, and the decrease of inflammation-related cells, TGF-β, and myeloperoxidase suggested a reduction in inflammation of the tissue surrounding the implant. Kyle et al. used an innovative maskless 3D grayscale manufacturing process to accurately replicate the layered micro- and nano-scale features of the acellular dermal matrix (ADM) on the surface of PDMS (97). The biomimetic morphology cues in ADM-modified silicone could alleviate the acute foreign body response of breast fibroblasts *in vitro*. Barr et al. performed statistical analysis on the natural surface of human breast tissue and constructed the first bionic breast tissue-derived breast implant surface using 3D grayscale lithography and ion etching technology (98). It showed that improving the implant material on the surface of the breast implant could reduce the inflammation of the implant-driven foreign body response.

## 7 Conclusion and Perspectives

The epidemiology and pathogenic factors of BIA-ALCL still need in-depth and continuous exploration. Within Asia, four cases of BIA-ALCL were reported in 2020 and 2021, confirming that the Asian population is not excluded from the risk. Moreover, due to the influence of various implant brands, individual patient differences, different surgical methods, and follow-up frequency in later stages, the actual incidence and detailed mechanisms of BIA-ALCL are worth considering. Secondly, in the presence of suspected ALCL features, detection methods based on pathology and imaging can be used synergistically for the comprehensive differential diagnosis of BIA-ALCL, which needs to be distinguished from the diagnosis of sALCL with similar characteristics. Especially, BIA-ALCL is a relatively uncommon disease with variable and atypical features in clinical, radiological, and pathological manifestations. Considering its susceptibility to being overlooked in diagnosis and the complexity and diversity of subsequent treatments, the multidisciplinary team (MDT) plays an important role in the diagnosis and treatment of patients with BIA-ALCL. Surgeons, oncologists, radiologists, and pathologists each provide independent medical opinions to patients. The management of these patients is from multiple dimensions to ensure that patients receive the optimal treatment and support. Thirdly, we have previously discussed the possible impact of the material and texture of the implant on ALCL. Notably, the nanomaterials provide an attractive strategy to resolve key issues related to breast implant-based implants, particularly in terms of biocompatibility, antimicrobial implantation, service life extension, and mechanical strength increase to address implant-related challenges. Nanomaterial modifications of existing breast implants are most promising for long-term implantation strategies. Functional nanomaterials may open up new dimensions for the future of breast implants. To achieve the unification of breast aesthetics and safety, especially in reducing the incidence of BIA-ALCL, the beneficial effects of implant modification still need to be confirmed by large-scale and long-term follow-up clinical trials. With the exposure of malignant tumors related to breast implants represented by BIA-ALCL, it is necessary to arouse enough awareness, but on the other hand, there is no need to cause too much panic in clinical practice.

## Author Contributions

YWa and QZ performed the literature investigation and wrote the manuscript. MW, YR, NZ, and YWu conceived the project and designed the outline. YT, WL, CZ, MX, and KH edited and revised the article. All authors contributed to the article and approved the submitted version.

## Funding

This work was supported by China Guanghua Science and Technology Foundation (grant 2019JZXM001) and Wuhan Science and Technology Bureau (grant 2020020601012241).

## Conflict of Interest

The authors declare that the research was conducted in the absence of any commercial or financial relationships that could be construed as a potential conflict of interest.

## Publisher’s Note

All claims expressed in this article are solely those of the authors and do not necessarily represent those of their affiliated organizations, or those of the publisher, the editors and the reviewers. Any product that may be evaluated in this article, or claim that may be made by its manufacturer, is not guaranteed or endorsed by the publisher.

## Abbreviations

2-methacryloxyethyl phosphorylcholine (PMPC); Acellular dermal matrix (ADM); Alginic acid (AA); Anaplastic large cell lymphoma (ALCL); Anaplastic lymphoma kinase (ALK); Bacterial biofilm (BF); Bovine albumin (BSA); Breast cancer (BC); Breast implant-associated anaplastic large cell lymphoma (BIA-ALCL); Carboxymethyl cellulose (CMC); Carboxymethyl β-1,3-dextran (CMD); C-C motif chemokine ligand 18 (CCL18); C-C motif chemokine receptor 6 (CCR6); Chitosan (CHI); Chromodomain helicase DNA binding protein 2 (CHD2); Chromosomal copy number aberrations (CNAS); Computed tomography (CT); Camp-response element binding protein (CREB); CREB binding protein (CREBBP); C-X-C motif chemokine ligand 14 (CXCL14); Dendritic cells (DCs); DNA methyltransferase 3 alpha (DNMT3A); Extracellular matrix (ECM); Fine needle aspiration (FNA); Flow cytometry (FCM); Fludeoxyglucose (FDG); Forward/side scatter (FSC/SSC); GATA binding protein 3 (GATA3); Graphene oxide (GO); Hyaluronic acid (HA); Human leukocyte antigen (HLA); Carbonic anhydrase 9 (CA9); Immunohistochemistry (IHC); Interferon-gamma (IFN-γ); Interleukin-10 (IL-10); Interleukin-2 (IL-2); Interleukin-3 (IL-3); Interleukin-6 (IL-6); Interleukin-13 (IL-13); Junb proto-oncogene (JunB); Layer-by-layer assembly (LbL); Li Fraumeni syndrome (LFS); Lysine methyltransferase 2C (KMT2C); Lysine methyltransferase 2D (KMT2D); Magnetic resonance imaging (MRI); Methicillin-resistant Staphylococcus aureus (MRSA); Nanoparticles (NPs); National Comprehensive Cancer Network (NCCN); Nitric oxide; Nϵ-myristoyl-lysine methyl ester (MKM); Photodynamic therapy (PDT); Platelet-derived growth factor receptor alpha (PDGFRA); Polydimethylsiloxanes (PDMS); Poly-l-lysine (PLL); Polymerase chain reaction (PCR); Positron emission tomography/computed tomography (PET/CT); Primary cutaneous ALCL (pcALCL); Programmed cell death 1 ligand 1 (PD-L1); Programmed cell death protein-1 (PD-1); Progression-free survival (PFS); phosphatidylinositol-4,5-bisphosphate 3-kinase catalytic subunit alpha (PIK3CA); Silicone rubber (SR); Special AT-rich sequence binding protein 1 (SATB1); Suppressor of cytokine signaling 1 (SOCS1); Suppressor of cytokine signaling 3 (SOCS3); Systemic ALCL (sALCL); T cell receptor (TCR); T helper cell 1 (Th1); T helper cell 17 (Th17); Titanium dioxide (TiO2) TNF receptor superfamily member 11a (TNFRSF11A); Tumor protein p53 (TP53); World Health Organization (WHO); Zinc Oxide (ZnO).

## Glossary

**Table:**

PMPC	2-methacryloxyethyl phosphorylcholine
ADM	Acellular dermal matrix
AA	Alginic acid
ALCL	Anaplastic large cell lymphoma
ALK	Anaplastic lymphoma kinase
BF	Bacterial biofilm
BSA	Bovine albumin
BC	Breast cancer
BIA-ALCL	Breast implant-associated anaplastic large cell lymphoma
CMC	Carboxymethyl cellulose
CMD	Carboxymethyl β-1,3-dextran
CCL18	C-C motif chemokine ligand 18
CCR6	C-C motif chemokine receptor 6
CHI	Chitosan
CHD2	Chromodomain helicase DNA binding protein 2
CNAS	Chromosomal copy number aberrations
CT	Computed tomography
CREB	Camp-response element binding protein
CREBBP	CREB binding protein
CXCL14	C-X-C motif chemokine ligand 14
DCs	Dendritic cells
DNMT3A	DNA methyltransferase 3 alpha
ECM	Extracellular matrix
FNA	Fine needle aspiration
FCM	Flow cytometry
FDG	Fludeoxyglucose
FSC/SSC	Forward/side scatter
GATA3	GATA binding protein 3
GO	Graphene oxide
HA	Hyaluronic acid
HLA	Human leukocyte antigen
CA9	Carbonic anhydrase 9
IHC	Immunohistochemistry
IFN-γ	Interferon-gamma
IL-10	Interleukin-10
IL-2	Interleukin-2
IL-3	Interleukin-3
IL-6	Interleukin-6
IL-13	Interleukin-13
JunB	Junb proto-oncogene
LbL	Layer-by-layer assembly
LFS	Li Fraumeni syndrome
KMT2C	Lysine methyltransferase 2C
KMT2D	Lysine methyltransferase 2D
MRI	Magnetic resonance imaging
MRSA	Methicillin-resistant Staphylococcus aureus
NPs	Nanoparticles
NCCN	National Comprehensive Cancer Network
MKM	Nitric oxide; Nϵ-myristoyl-lysine methyl ester
PDT	Photodynamic therapy
PDGFRA	Platelet-derived growth factor receptor alpha
PDMS	Polydimethylsiloxanes
PLL	Poly-l-lysine
PCR	Polymerase chain reaction
PET/CT	Positron emission tomography/computed tomography
pcALCL	Primary cutaneous ALCL
PD-L1	Programmed cell death 1 ligand 1
PD-1	Programmed cell death protein-1
PFS	Progression-free survival
PIK3CA	phosphatidylinositol-4,5-bisphosphate 3-kinase catalytic subunit alpha
SR	Silicone rubber
SATB1	Special AT-rich sequence binding protein 1
SOCS1	Suppressor of cytokine signaling 1
SOCS3	Suppressor of cytokine signaling 3
sALCL	Systemic ALCL
TCR	T cell receptor
Th1	T helper cell 1
Th17	T helper cell 17
TiO2	Titanium dioxide
TNFRSF11A	TNF receptor superfamily member 11a
TP53	Tumor protein p53
WHO	World Health Organization
ZnO	Zinc Oxide
